# Potential mechanisms of Lian-Zhi-Fan solution for TNBS-induced ulcerative colitis in rats *via* a metabolomics approach

**DOI:** 10.3389/fphar.2022.1014117

**Published:** 2022-12-02

**Authors:** Junyi Bai, Tingting Xiong, Xiao Wang, Yanfen Cheng, Ruifeng Luo, Xiangdong Yang, Chaomei Fu

**Affiliations:** ^1^ State Key Laboratory of Southwestern Chinese Medicine Resources, Pharmacy School, Chengdu University of Traditional Chinese Medicine, Chengdu, China; ^2^ Chengdu Anorectal Hospital, Chengdu, China; ^3^ Sichuan Provincial Maternity and Child Health Care Hospital, Chengdu, China

**Keywords:** ulcerative colitis, metabolomics, HPLC/Q-TOF-MS, TNBS, rectal administration

## Abstract

Lian-Zhi-Fan (LZF) decoction is a hospital-prescribed traditional Chinese medicine botanical drug prepared by the fermentation of decocted Coptidis Rhizome (Huanglian), Gardeniae Fructus (Zhizi), and alum (Baifan). It has been used clinically in China for the treatment of anal fistula, perianal abscess, ulcerative colitis (UC), and other anorectal diseases for hundreds of years. However, due to the complexity of traditional Chinese medicine, the potential mechanisms of LZF in the treatment of UC have remained unknown. This study primarily investigated the remarkable pharmacological effects of LZF on TNBS-induced UC rats. To explore the complex targets and regulatory mechanisms of metabolic networks under LZF intervention, a metabolomics approach mediated by HPLC/Q-TOF-MS analysis was used to screen the different metabolites and their metabolic pathways in the serum in order to characterize the possible anti-UC mechanisms of LZF. After rectal administration of LZF for seven consecutive days, significant amelioration effects on body weight loss, DAI score, and colon inflammation were found in UC rats. Based on this, further metabolomics identified 14 potential biomarkers in the treatment of UC with LZF, of which five possessed diagnostic significance: L-alanine, taurocholic acid, niacinamide, cholic acid, and L-valine. These metabolites are mainly involved in 12 metabolic pathways, including nicotate and nicotinamide metabolism, glycospholipid metabolism, arginine and proline metabolism, primary bile acid biosynthesis, and pantothenate and CoA biosynthesis. These metabolic pathways suggest that LZF ameliorates UC by regulating amino acid metabolism, fat metabolism, and energy production. This study provides a useful approach for exploring the potential mechanisms of herbal prescription in UC treatment mediated by metabolomics.

## 1 Introduction

Ulcerative colitis (UC) is a nonspecific inflammatory disease, with persistent or recurrent abdominal pain, diarrhea, and purulent mucinous bloody stool as its main clinical manifestations. It can extend from the rectum to the proximal colon, and it is one of the chronic inflammatory bowel diseases (IBDs) (Ungaro et al., 2017). UC has the characteristics of early onset, easy recurrence, long course of disease, and increased cancer risk, resulting in poor quality of life and high economic burden (Hoivik et al., 2013). In recent years, the incidence rate of UC has been high in developed countries but has tended to be stable. Moreover, it has been increasing year by year in developing countries. Therefore, UC has become a refractory disease worldwide (Alatab et al., 2020). The exact pathogenesis of UC is not completely clear, but it is related to immune disorder, intestinal microbiota imbalance, genetic susceptibility, environment, and other factors (Kobayashi et al., 2020). At present, drug-graded treatment is often implemented in clinic, according to the severity of UC: 5-aminosalicylic acid is selected as the first-line drug in mild and moderate cases and immunosuppressive or biological drugs in moderate and severe cases. In addition, corticosteroids and fecal transplantation can be used to treat acute UC. However, these treatments have limited efficacy and obvious side effects. Therefore, the use of alternative therapies with high efficiency and low side effects in traditional Chinese medicine (TCM) has been a breakthrough in UC treatment and has attracted extensive attention (Zhang et al., 2013; He et al., 2022).

Lian-Zhi-Fan (LZF) decoction (z20080191) is a hospital-grade preparation used at Chengdu Anorectal Hospital. It is a liquid prepared from the fermentation of Coptidis Rhizome (Huanglian), Gardeniae Fructus (Zhizi), and alum (Baifan). In China, it has been used for hundreds of years because of its remarkable clinical effects. It is commonly used in the prevention and treatment of anorectal diseases such as anal fistula, anal condyloma acuminatum, anal dilatation, perianal abscess, and chronic ulcerative proctitis. LZF contains a variety of effective components for the treatment of UC, including berberine, coptisine, gardenoside, and genipin. Berberine, the representative component of Coptidis Rhizome, could effectively improve UC by reducing inflammation, regulating immunity, regulating the intestinal flora, and protecting the intestinal barrier (Li et al., 2016; Li et al., 2020; Liao et al., 2020). Geniposide, a representative component of Gardeniae Fructus, could improve DSS-induced UC by inhibiting oxidative stress, reducing inflammation, and protecting the intestinal barrier (Zhang et al., 2017; Yang et al., 2020; Lu et al., 2021). Deepening research into the mechanism of the compounds contained in LZF for improving UC has revealed the mechanism of LZF as an effective therapeutic for UC to a certain extent. However, in view of this complex formulation, there is no detailed study of the mechanism of action of LZF in improving UC.

Metabolomics reflects the relationship between the state of the biological system and the concentration of small-molecule metabolites, and it characterizes the overall metabolic spectrum in complex biological matrixes. It is especially suitable for characterizing the mechanism of action of a variety of stimulating factors on the whole body. Therefore, it is increasingly used to study the mechanism of TCM in improving diseases (Wang et al., 2017; Wang et al., 2019). Metabolomics was used to find that Xiaokeyinshui extract combination improves the symptoms of Type 2 diabetes mellitus mice by regulating glucose metabolism, lipid metabolism, and amino acid metabolism (Xiang et al., 2021). Metabolomics has shown that Huang-Lian-Jie-Du decoction reduces acute UC in mice by regulating arachidonic acid metabolism and glycerophospholipid metabolism (Yuan et al., 2020). Metabolomics technology was also used to discover and study changes in 36 potential biomarkers upon compound sophorae decoction treatment in UC rats, revealing the mechanism of this prescription in improving UC (Hong et al., 2021). Obviously, the wide application of metabolomics technology has provided a new perspective for the study of the mechanism of action of TCM.

In this study, the improvement effects of LZF on TNBS-induced UC in rats was studied based on the metabolomics of HPLC/Q-TOF-MS. The anti-colitis effect of LZF was evaluated by colon length, body weight change, DAI, and H&E pathological sections. Metabolomic analysis was performed on serum samples of rats to screen potential marker metabolites and evaluate their clinical diagnostic value, enrich metabolic pathways, and reveal the mechanism of LZF in improving UC from a metabolic perspective.

## 2 Materials and methods

### 2.1 Chemicals and animal

TNBS (5%) was purchased from Sigma-Aldrich (United States). LZF was purchased from Chengdu Anorectal Hospital (Chengdu, China). Ethanol was purchased from Chengdu Kelong Chemical Reagent Factory (Chengdu, China). Paraformaldehyde was purchased from Sigma-Aldrich (United States). Sodium chloride injection was obtained from Sichuan Kelun Pharmaceutical Co., Ltd. (Chengdu, China).

Male Sprague–Dawley rats (180–200 g) were obtained from Chengdu Dossy Biological Technology Co., Ltd. (Chengdu, China). The rats were housed under standard conditions (temperature 20–23°C, humidity 50 ± 10%, 12 h light/dark cycles) and supplied *ad libitum* with food and distilled water. All animal studies were conducted and performed as per the protocol approved by the Animal Welfare Committee of Chengdu University of Traditional Chinese Medicine.

### 2.2 Composition of LZF solution

The LZF solution (1000 ml) was prepared by fermentation and filtration of 43.5 g *Coptis chinensis* Franch [*Ranunculaceae*; Coptidis Rhizoma], 31.5 g *Gardenia jasminoides* J. Ellis [*Rubiaceae*; Gardeniae Fructus], and 4.35 g alum [KAl(SO_4_)_2_·12H_2_O].

### 2.3 Preparation of LZF solution

LZF was prepared according to the standard of LZF solution (approved by Sichuan Food and Drug Administrations, zbz20080900). Huanglian (43.5 g) and Zhizi (31.5 g) were crushed, soaked in water for 24 h, and decocted three times. The same amount of alum was added during each decocting, with a total of 4.35 g added. In the first decocting, eight times the amount of water was added and decocted for 45 min. In the second and third decoctions, six times the amount of water was added and decocted for 30 min. The decoctions were combined and concentrated to 1000 ml and placed at room temperature for fermentation for three weeks, then filtered to obtain LZF.

### 2.4 Animal treatment

The SD rats were randomly divided into four groups (six rats in each group): the control group, model group, LZF-L group, and LZF-H group. After adaptive feeding, the rats in the control group were given 2 ml of 0.9% normal saline through the rectum, and rats in other groups were given TNBS-solution (30 mg/kg, soluble in 50% ethanol) inserted to 8 cm from the anus to induce UC (Yang et al., 2017). After successful modeling, the rats in the control and model groups were rectally given 2 ml normal saline, the rats in the LZF-L group were rectally given LZF (0.4 g/kg, crude botanical drug), and the rats in LZF-H group were rectally given LZF solution (0.8 g/kg, crude botanical drug). All groups were rectally given the corresponding solutions continuously for seven days. During the whole experiment, the body weight and disease activity index (DAI) score were recorded daily. DAI was calculated as the sum of weight loss (0 = <5%, 1 = 5–10%, 2 = 10–15%, 3 = 15–20%, and 4 >20%); fecal bleeding (0 = none, 1 = mild occult blood, 2 = severe occult blood, 3 = visible blood, and 4 = major bleeding); and stool consistency status (0 = normal, 1 = soft, 2 = loose stools, 3 = diarrhea, and 4 = severe diarrhea) (Zhang et al., 2022a; Zhang et al., 2022b).

### 2.5 Sample collection and preparation

After fasting for 24 h following the final administration, the rats were anesthetized with sodium pentobarbital, and their blood was collected from the abdominal aorta. Colon tissue was obtained by dissection, and the length was measured. After the blood was coagulated at 4°C for 2 h, it was centrifuged at 3500 rpm for 10 min, and its supernatant was transferred to a centrifuge tube and immediately stored at −80°C until analysis.

### 2.6 Histological evaluation

After colon tissue was fixed with 10% formalin, it was embedded in paraffin, cut into 5-μm sections, and stained with hematoxylin and eosin (H&E) (Wang et al., 2021). Images were collected using an optical microscope.

### 2.7 Metabolic analysis of serum samples

#### 2.7.1 Preparation of serum samples

Serum samples were thawed at 4°C, and 100 μL sample was put into a centrifuge tube with 400 μL of methanol, vortexed for 60 min, and centrifuged at 12,000 rpm for 10 min at 4°C. The supernatant was filtered through a 0.22-μm membrane and analyzed by HPLC/Q-TOF-MS.

A volume of 20 μL of each sample was removed and mixed to obtain a quality control sample to eliminate systematic errors in the experiment.

#### 2.7.2 HPLC/Q-TOF-MS conditions

All analyses were carried out on the Agilent 1200 HPLC instrument (Agilent, Germany). The chromatographic separation was performed on an ACE Excel C18 column (100 mm × 2.1 mm, 3.0 mm) at 40°C. The mobile phases were composed of (A) 0.1% (v/v) formic acid aqueous solution and (B) acetonitrile containing 0.1% formic acid with a flow rate of 0.4 ml/min. The gradient elution program for serum samples was carried out as follows: 0–2 min, 5% B; 2–7 min, 5–65% B; 7–20 min, 65–95% B; 20–22 min, 95% B; 22–23 min, 95–5% B; and 23–28 min, 5% B. The sample injection volume was set to 3 μL.

Mass spectrometry analysis was conducted to confirm the peak identities using the Agilent 6530 Q-TOF mass spectrometer (Agilent Corp, United States) coupled with an electrospray ionization (ESI) source. MassHunter Workstation software (Agilent Technologies, United States) was employed for the system operation. The operation conditions of the mass spectrometer were as follows: capillary voltage of 4.0 kV for the positive ion mode and 3.5 kV for the negative ion mode; nebulizer pressure of 35 psig; gas temperature of 320°C; gas flow of 12 L/min; collision energy of 35 eV; drying gas temperature of 300°C; and drying gas flow rate of 6 L/min. The mass range was set from 50 to 1100 Da with the full scan mode.

#### 2.7.3 Data processing and multivariate analysis

The initial HPLC/Q-TOF-MS data were pre-processed with Peak Picking and Threshold Peak Filer in MSConvert (version 3.0, ProteoWizard, United States) to generate mzXML documents. Then the document was imported into XCMS website software (https://xcmsonline.scripps.edu) for noise removal, baseline correction, and peak alignment and to automatically generate a TSV document with a multi-dimensional data matrix composed of retention time, peak intensity, and mass-to-charge ratio (m/z). The final peak areas in the TSV files were re-obtained using the 80% correction method.

The pre-treatment data files were imported into the SIMCA (ver. 14.0) software package (Umetrics, Umea, Sweden) for multivariate statistical analyses including PCA, PLS-DA, and OPLS-DA. Differences between groups were explored based on endogenous metabolites, and variable importance in projection (VIP) values >1.5 were selected to prove the contributions to variation (Sun et al., 2018). These differential metabolites were considered as potential chemical markers. Then they were further identified *via* GraphPad Prism software (version 9.0, GraphPad, United States) and analyzed with a one-way ANOVA test to evaluate significant differences between the groups (*p* < 0.05).

#### 2.7.4 Biomarker identification and pathway analysis

The screening of potential metabolites was based on the retention time, the accurate quality of MS and MS/MS, and the results of comparison against various databases, such as the Human Metabolome Database (http://www.hmdb.ca) and the Kyoto Encyclopedia of Genes and Genomes (http://www.genome.jp/kegg/). Various metabolic pathways of biomarkers were further analyzed *via* the MetaboAnalyst database (http://www.metaboanalyst.ca/) to obtain the enrichment analysis and biological functions of different metabolites.

### 2.8 Statistical analysis

Data were presented as mean ± SD. Statistical comparisons were assessed by one-way ANOVA test between different groups using GraphPad Prism (version 9.0, GraphPad, United States). Values of *p < 0.05 were regarded as indicating significant differences.

## 3 Results

### 3.1 Therapeutic effects of LZF on UC rats

**FIGURE 1:**
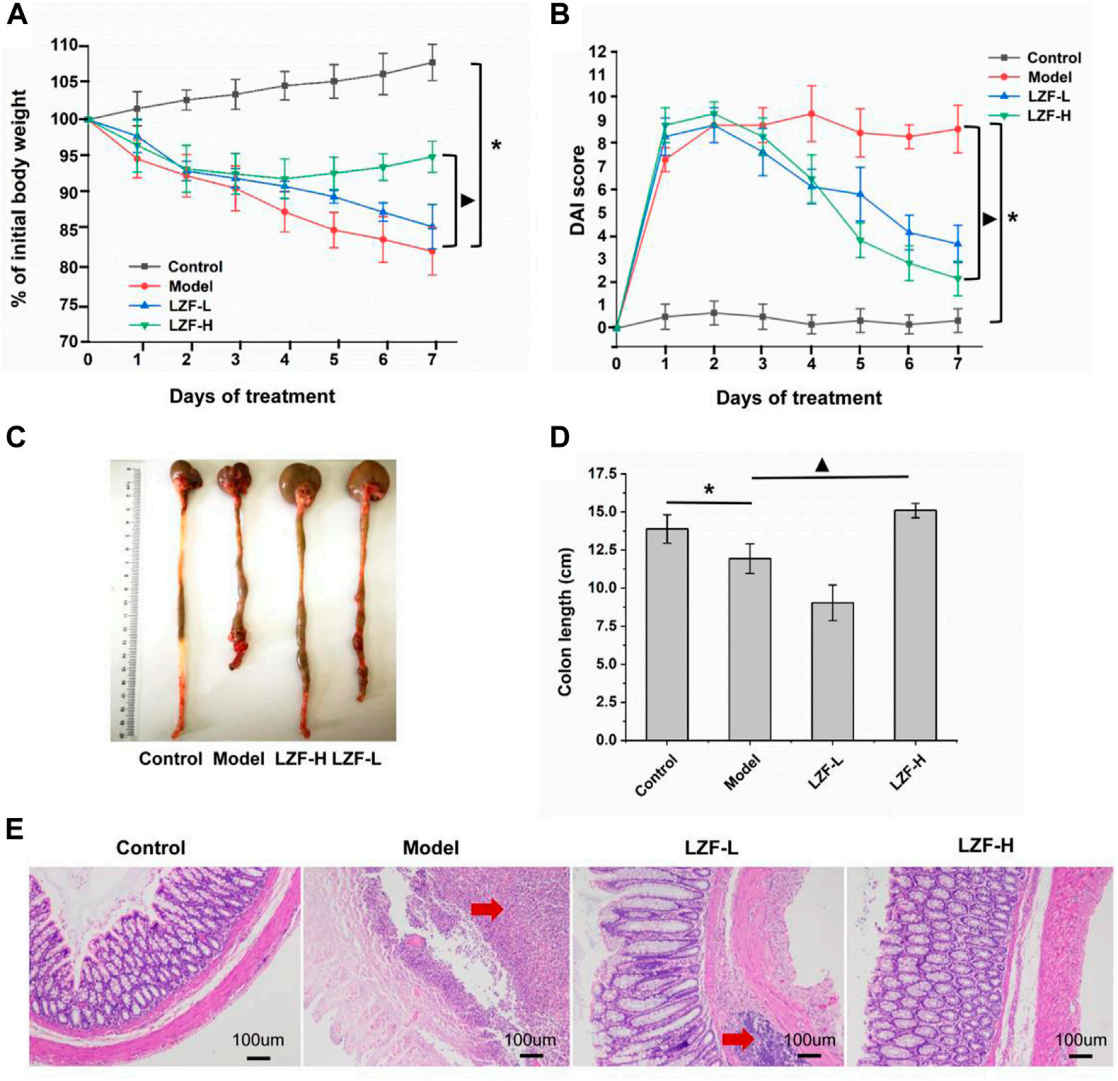
Therapeutic effect of LZF solution on ulcerative colitis caused by TNBS. **(A)** Body weight; **(B)** DAI score; **(C)** colon image; **(D)** colon length; and **(E)** histopathological section of colon.

The clinical symptoms of UC often include diarrhea, bloody stool, and weight loss. Grading the severity of these three items to form the DAI score has become an important index in drug treatment of colitis (Wang et al., 2021). In addition, colon length intuitively reflects the degree of colon lesions, which are important detection indicators (Yu T et al., 2021). As shown in Figure 1A, compared with the control group, the weight of rats in the model group induced by TNBS decreased significantly (*p* < 0.05), and LZF significantly alleviated the trend of weight loss. The LZF-H group showed the strongest effect, which was significantly different from the model group. Figure 1B shows the DAI results of each group. The DAI was about 0 for the control group and continued at 8–10 for the model group, while LZF continued to reduce the DAI from the second day, with the LZF-H group decreasing most significantly compared to the model group (*p* < 0.05). Figures 1C,D show the appearance and length of the colon in each group; colon length results, showing that the model group had the shortest colon; and that the LZF-H group effectively inhibited the colon shortening caused by TNBS, with colons restored to normal length. In conclusion, the results of colitis-related indicators, including colon length, body weight, and DAI score, showed that LZF could significantly improve the UC caused by TNBS and that the therapeutic effect increased with dose.

### 3.2 Results of colonic tissue sections stained by H&E

H&E staining sections were used to observe the infiltration of inflammatory cells in colon tissue by comparing the morphology and size to reflect reduced inflammation in colon tissue (Zhang et al., 2021). The results of tissue sections (Figure 1E) showed that the structure of intestinal mucosa in the normal group was complete: the intestinal crypts were arranged orderly, and there was no edema in the submucosa. However, in the model group, the intestinal mucosa fell off, many inflammatory cells infiltrated, and the structure of intestinal recesses was seriously damaged. In the LZF-L group, the mucosa partially fell off and the recess structure remained intact, but there was inflammatory cell infiltration and obvious edema in the submucosa. In LZF-H group, the mucosal tissue fell off slightly, the recess structure was complete, and there was no edema in the submucosa. The microstructure of colon tissue showed that LZF could protect the normal physiological morphology of the colon and provide an intuitive basis for improving the effects of colitis.

### 3.3 Multivariate analysis of HPLC-QTOF-MS data

**FIGURE 2:**
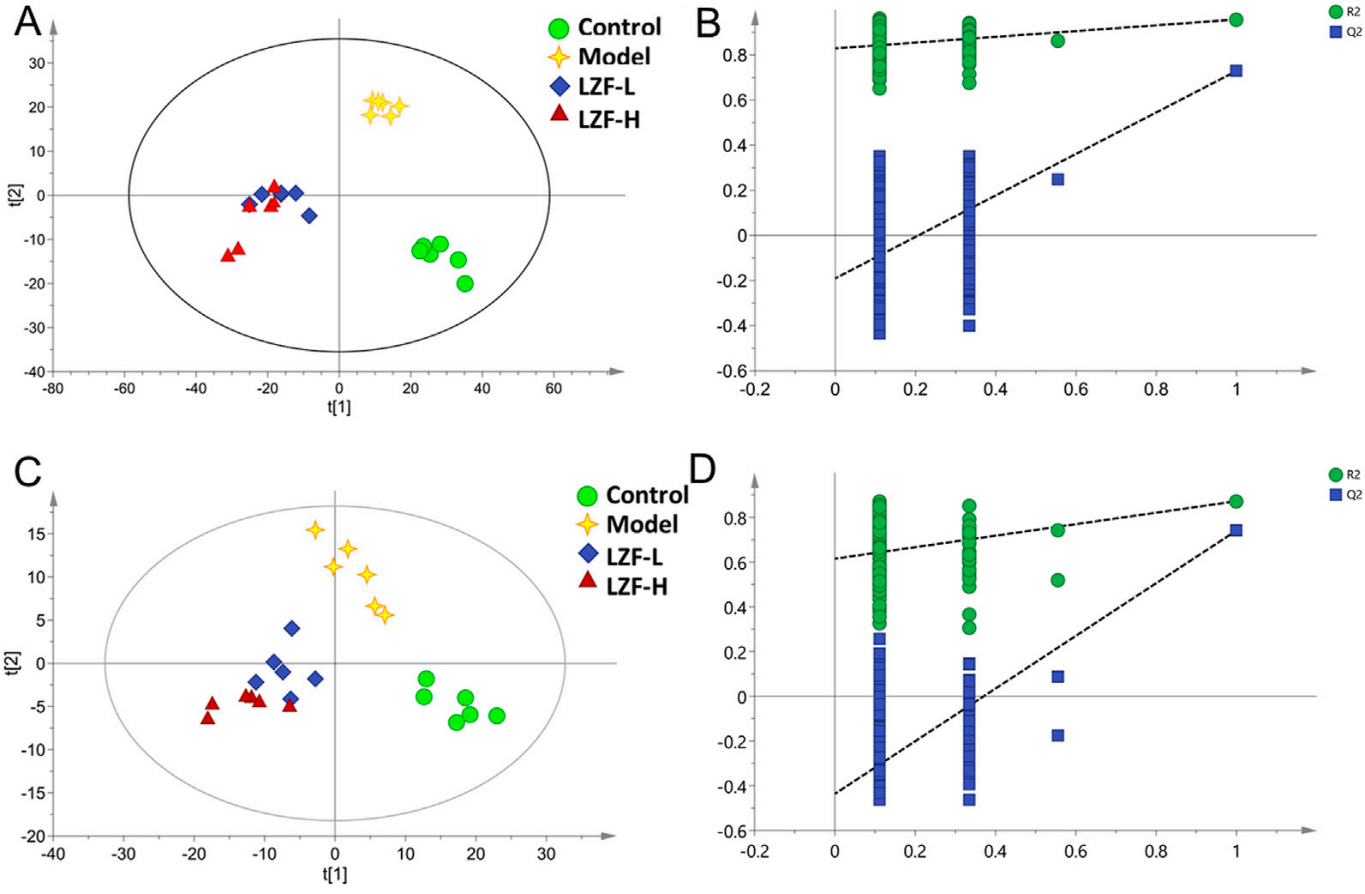
Multivariate statistical analysis of serum mass spectrometry. **(A)** PLS-DA score plot in the positive ion mode; **(B)** 200 permutation tests of the PLS-DA score plot in the positive ion mode; **(C)** PLS-DA score plot in the negative ion mode; and **(D)** 200 permutation tests of the PLS-DA score plot in the negative ion mode.

Mass spectrum information for serum samples in each group was obtained in positive and negative ion modes by HPLC-QTOF-MS. To find visual differences among the four groups, the multidimensional data after normalization, retention time correction, and peak picking were imported into SIMCA 14.0 software for multivariate statistical analysis. An overview of serum metabolic profiling was initially performed by a supervised PLS-DA analysis, which obtained more ideal intergroup separation and raised the identification of variables for the classification. To judge the reliability and accuracy of the model, PLS-DA score plots and 200 permutation test methods were provided for evaluation, as shown in Figures 2A–D. Among them, in the positive ion mode (Figure 2C), R2X = 0.384, R2Y = 0.948, and Q2 = 0.602; and in the negative ion mode (Figure 2D), R2X = 0.447, R2Y = 0.911, Q2 = 0.601, indicating that the model had not been over-fitted. The PLS-DA analysis indicated that serum samples for the control, model, LZF-L, and LZF-H groups showed clearly different classifications and separation in both positive and negative ion modes. The LZF-L and LZF-H groups were gradually closer to the normal group than to the model group. The TNBS-induced UC model could be considered successful. Therefore, these results show that the serum samples of each group had good separation, which is conducive to the further screening of differential metabolites.

To further differentiate metabolites between the two groups, OPLS-DA was used to better discriminate among the control and model groups, the model and LZF-L groups, and the model and LZF-H groups in both positive- and negative-ion modes. Based on the VIP) value >1, higher VIP values of metabolites indicated that these variables made significant contributions to the separation of each group. These results show that the OPLS-DA model exhibits favorable fitness and prediction.

### 3.4 Identification of structural elucidation of differential biomarkers

**FIGURE 3:**
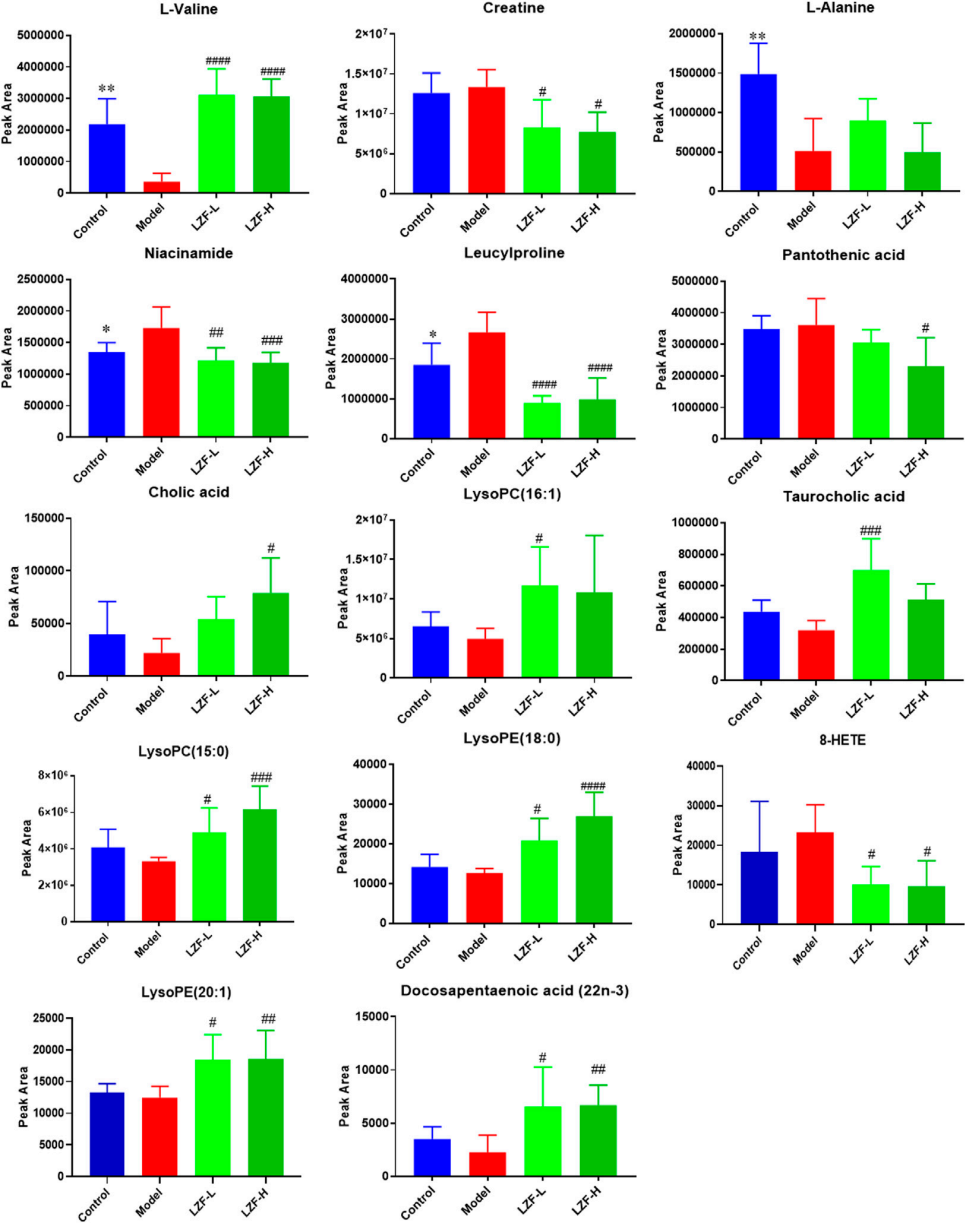
Peak area analysis of 14 differential metabolites (*p < 0.05, **p < 0.01, ***p < 0.001, control group vs. model control; ^#^ *p* < 0.05, ^##^ *p* < 0.01, ^###^ *p* < 0.001 low-dose, LZF solution group vs. model control).

According to the VIP score of OPLS-DA for the samples in the aforementioned groups, combined with one-way ANOVA tests performed by SPSS software while the ions satisfying VIP ≥ 1 and *p* ≤ 0.05 were screened out, potential biomarkers were identified. A total of 35 metabolite ions with high VIP values were selected and isolated from serum samples for further investigation. After a one-way ANOVA test based on accurate molecular mass measurement, fragmentation ions, peak mass spectrum data, and molecular formula, only 14 (including amino acids, bile acids, fatty acids, peptides, and vitamins) were ultimately identified as potential biomarkers for control and model rats, as shown in Table 1. When the model group was adjusted up or down compared with the normal group, the LZF solution-treated group tended to call-back toward the normal group. Finally, the variation trend of these differential metabolites was analyzed using one-way ANOVA adjusted by the FDR method, as shown by the statistical histogram drawn with the peak areas of the four groups in Figure 3.

**TABLE 1:** Identified potential biomarkers of ulcerative colitis.

No.	Metabolite	Rt (min)	Determined mass (m/z)	Calculated mass (m/z)	Mass error (ppm)	Formula	Ion mode	VIP value	*q*-value
1	L-Valine	0.86	140.0681	140.0682	1	C5H11NO2	[M + Na]+	4.05	<0.0001
2	Creatine	0.88	132.0772	132.0768	3	C4H9N3O2	[M + H]+	2.15	0.0059
3	L-Alanine	0.88	90.0557	90.0550	8	C3H7NO2	[M + H]+	2.56	0.0018
4	Niacinamide	1.07	123.0554	123.0553	1	C6H6N2O	[M + H]+	1.75	0.0012
5	Leucylproline	1.50	229.1552	229.1547	2	C11H20N2O3	[M + H]+	3.35	0.0004
6	Pantothenic acid	2.09	220.1164	220.1179	7	C9H17NO5	[M + H]+	2.49	0.0156
7	Cholic acid	8.13	407.2768	407.2803	9	C24H40O5	[M-H]-	2.08	0.0237
8	LysoPC(16:1)	9.06	494.3224	494.3241	3	C24H48NO7P	[M + H]+	3.22	0.0471
9	Taurocholic acid	9.06	516.3033	516.2989	8	C26H45NO7S	[M + H]+	1.78	0.0078
10	LysoPC(15:0)	9.25	482.3217	482.3241	5	C23H48NO7P	[M + H]+	3.26	0.0011
11	LysoPE (18:0)	9.25	526.3118	526.3150	6	C23H48NO7P	[M + FA-H]-	1.41	<0.0001
12	8-HETE	9.95	319.2247	319.2279	10	C20H32O3	[M-H]-	3.64	0.0262
13	LysoPE (20:1)	10.05	506.3218	506.3252	7	C25H50NO7P	[M-H]-	1.07	0.0038
14	Docosapentaenoic acid (22n-3)	13.62	329.2472	329.2486	4	C22H34O2	[M-H]-	1.12	0.0311

**FIGURE 4:**
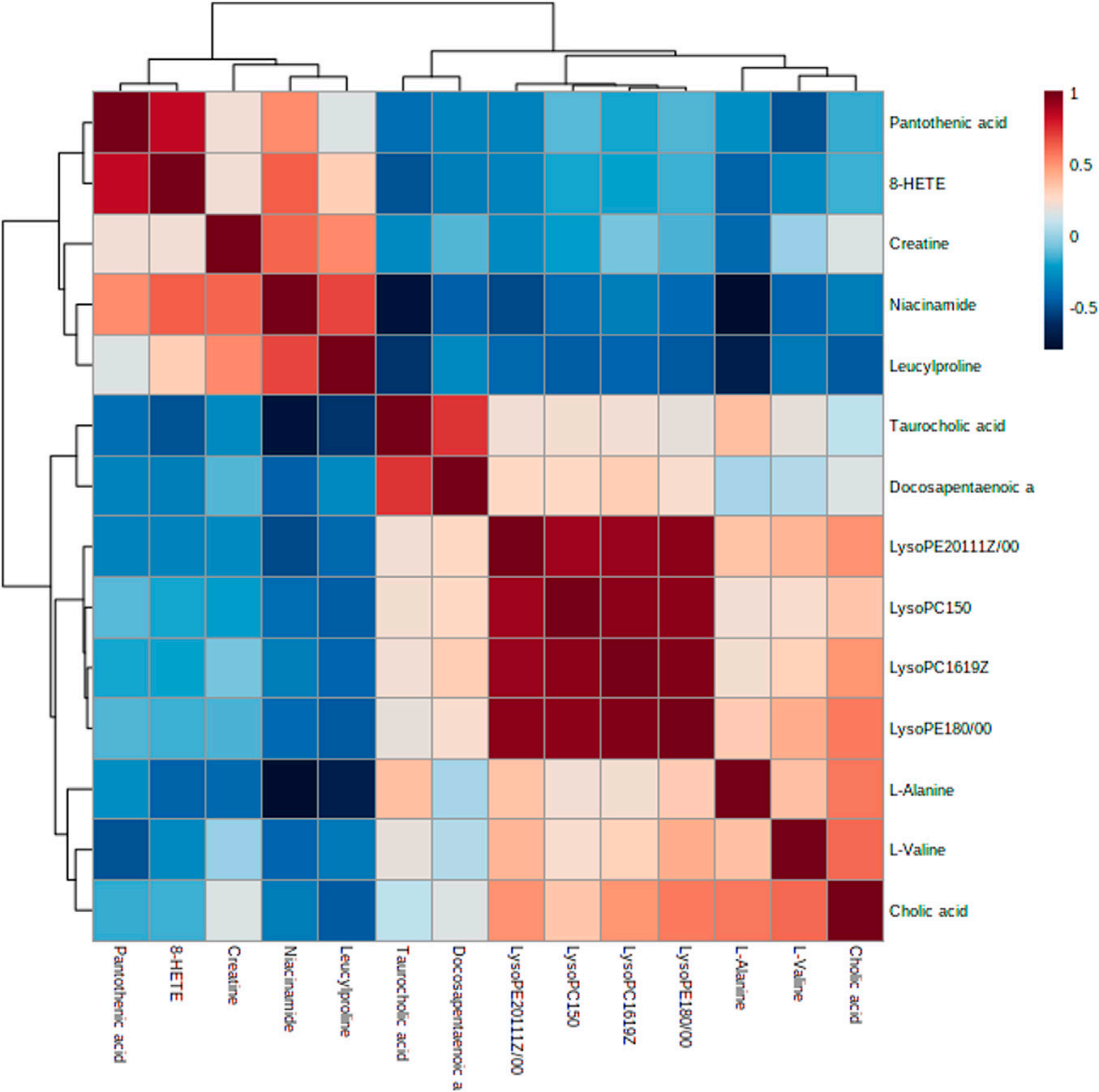
Correlation analysis of potential metabolite biomarkers in the LZF-L group compared to the model group.

Furthermore, combined with correlation analysis heat maps (in which blue indicates a negative correlation, red represents a positive correlation, and the color degree manifests the depth correlation), the change trends of different metabolites biomarkers were determined (Figure 4) between the model and LZF-L groups. Five metabolites—creatine, niacinamide, leucylproline, pantothenic acid, and 8-HETE—accumulate with an elevated trend in the model group, while the other metabolites accumulate in a decreasing trend in LZF-L group. These results are consistent with the aforementioned results.

### 3.5 Diagnostic value analysis of diversified ROC curves

**FIGURE 5:**
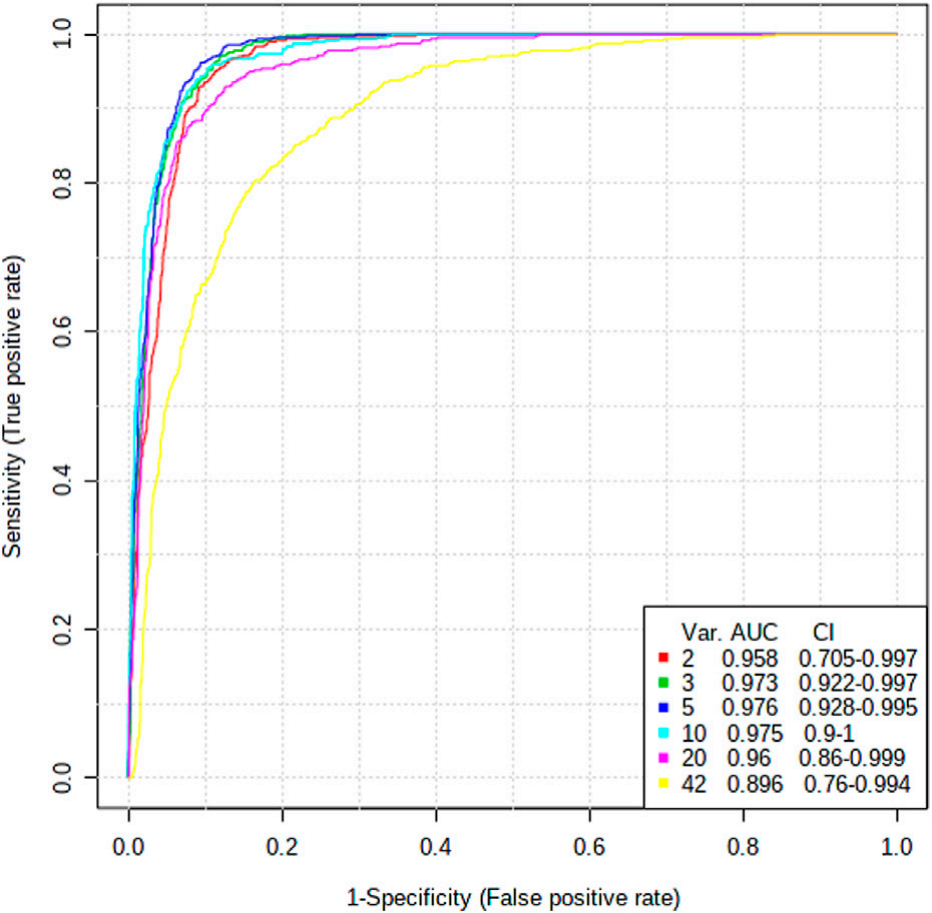
ROC curve analysis of 14 potential biomarkers between control and model groups.

Characterizing the clinical diagnosis value of potential metabolomics biomarkers is a major task, and the utilization of receiver operating characteristic (ROC) curves to evaluate the diagnostic accuracy of biomarkers between the two groups has been successful in many studies (Wu et al., 2010). Area under the curve (AUC) is used to evaluate the clinical value of ROC curves, with biomarkers with AUC values greater than 0.8 being highly accurate and obviously differential (Broadhurst and Kell, 2006). As shown in Figure 5, the AUC value is > 0.8, and the diagnostic sensitivity and specificity are high, indicating that these metabolites used as biomarkers for TNBS-induced UC disease diagnosis have high clinical diagnostic value. L-Alanine (AUC = 1.000), taurocholic acid (AUC = 0.917), niacinamide (AUC = 0.861), cholic acid (AUC = 0.861), and L-valine (AUC = 0.847) had AUC values higher than 0.8, suggesting that they can effectively contribute to TNBS-induced UC disease diagnosis.

### 3.6 Metabolic pathway analysis

**FIGURE 6:**
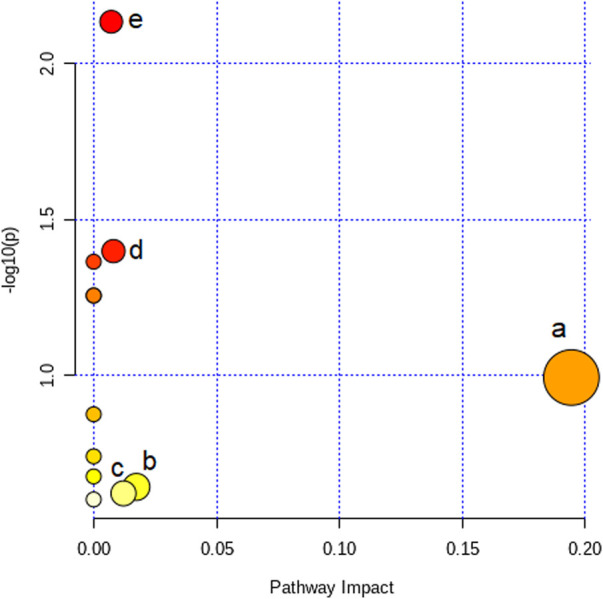
Impact value of metabolic pathway analysis (a. nicotinate and nicotinamide metabolism, b. glycerophospholipid metabolism, c. arginine and proline metabolism, d. primary bile acid biosynthesis, and e. pantothenate and CoA biosynthesis).

**FIGURE 7:**
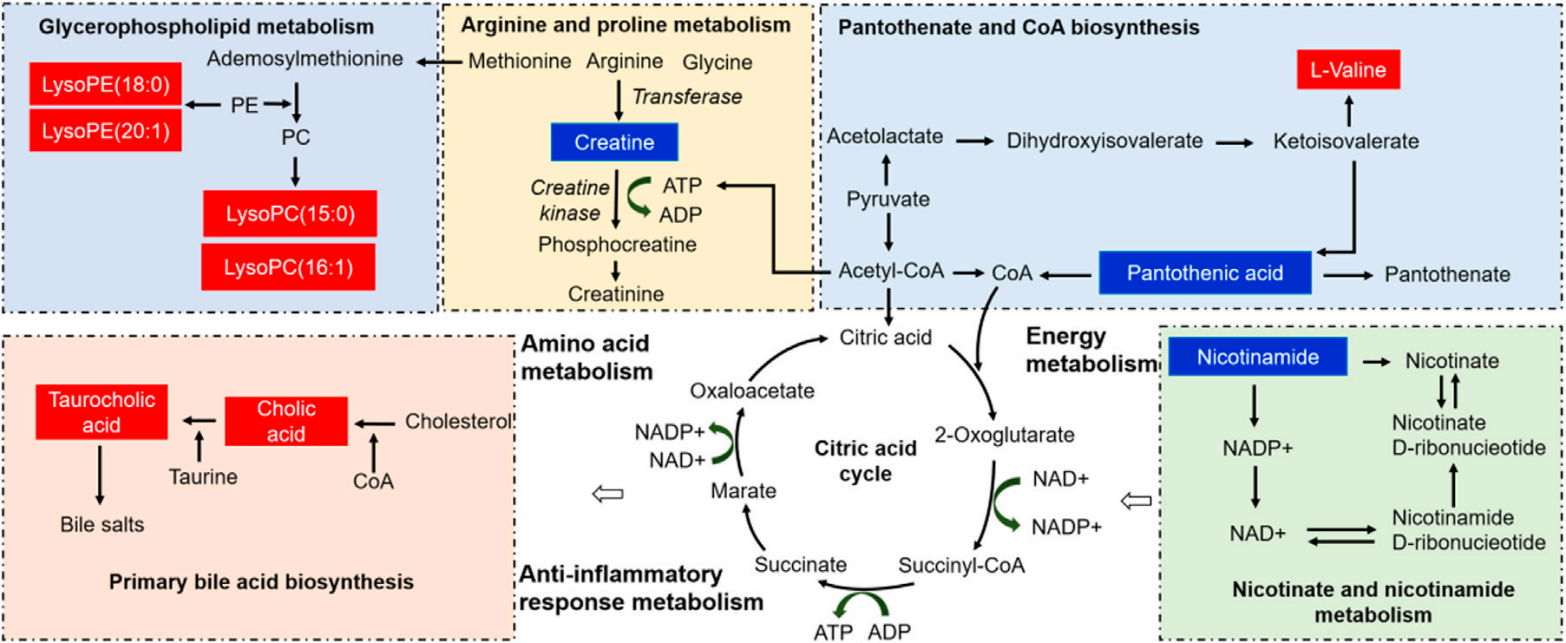
Correlation pathway networks of treatment with LZF solution in TNBS-induced UC. The red and blue represent the metabolites increased and decreased, respectively, compared with the model group. (PC: phosphatidylcholine, PE: phosphatidylethanolamine, LysoPC: lysophosphatidylcholine, LysoPE: lysophosphatidylethanolamine).

To analyze the TNBS-induced UC metabolic pathway, the identified biomarkers related to UC were imported into MetaboAnalyst 4.0 software to determine which metabolic pathways were perturbed by the modeling. Twelve disturbed metabolic pathways were detected with large impact values (>0.00) and significant *p*-values (<0.05), including nicotinate and nicotinamide metabolism, glycerophospholipid metabolism, arginine and proline metabolism, primary bile acid biosynthesis, and pantothenate and CoA biosynthesis, as shown in Figure 6 and Table 2. The aforementioned pathways may be the potential mechanisms underlying changes in endogenous substance metabolism after treatment with LZF. In addition, various metabolic pathways *in vivo* are interrelated, and LZF plays a synergistic role in multiple pathways. Therefore, the construction of a metabolic pathway interconnection network is conducive for describing the therapeutic mechanism of LZF solution in UC rats. As shown in Figure 7, the interaction of different metabolic pathways shows that UC is a disease involving multiple metabolic disorders that regulate amino acid metabolism, fat metabolism, and energy generation, while LZF could improve UC by regulating these metabolic pathways.

**TABLE 2:** Summary of metabolic pathways of potential biomarkers.

Metabolic pathway	Total	Hits	Raw *p*	-log (*p*)	Impact	No. in Figure 6
Pantothenate and CoA biosynthesis	19	2	0.0073339	2.1347	0.00714	e
Primary bile acid biosynthesis	46	2	0.039973	1.3982	0.00805	d
Aminoacyl-tRNA biosynthesis	48	2	0.043228	1.3642	0	—
Valine, leucine, and isoleucine biosynthesis	8	1	0.055506	1.2557	0	—
Taurine and hypotaurine metabolism	8	1	0.055506	1.2557	0	—
Nicotinate and nicotinamide metabolism	15	1	0.10176	0.99242	0.1943	a
Selenocompound metabolism	20	1	0.13353	0.87443	0	—
Alanine, aspartate, and glutamate metabolism	28	1	0.18224	0.73936	0	—
Glycine, serine, and threonine metabolism	33	1	0.2114	0.67489	0	—
Glycerophospholipid metabolism	36	1	0.22844	0.64122	0.01736	b
Arginine and proline metabolism	38	1	0.23962	0.62048	0.01212	c
Valine, leucine, and isoleucine degradation	40	1	0.25064	0.60094	0	—

## 4 Discussion

Metabolomics has proved its capability and feasibility to explore the pharmacodynamics of botanical drugs and to reveal the intricate mechanisms of interactions between medicines and organisms. This study verified the therapeutic effect of the classic prescription of LZF on TNBS-induced UC rats. First, it confirmed that LZF has a good therapeutic effect on UC rats according to body weight, DAI, colon length, and pathological sections. Furthermore, HPLC-QTOF-MS was used to explore the mechanism of LZF on UC rats. A total of 14 potential biomarkers for the treatment of UC were identified, including amino acids, bile acids, lipid species (LysoPCs/LysoPEs), peptides, and vitamins. LZF plays a key role in regulating five significant metabolic pathways (pantothenate and CoA biosynthesis, nicotinate and nicotinamide metabolism, primary bile acid biosynthesis, glycerophospholipid metabolism, and arginine and proline metabolism) by normalizing energy production, amino acids, and inflammation in metabolic disorders. The following is a discussion of the biological significance of the aforementioned potential molecules and five aspects of dominant pathways to further reveal the mechanism of action of LZF on UC rats.

Energy metabolism is the most basic organic means of providing energy for all metabolic activities (Yu Z.W. et al., 2021). The treatment of UC rats with LZF solution mainly includes two energy metabolic pathways: pantothenic acid and CoA biosynthesis and niacin and nicotinamide metabolism. Both pantothenic acid and L-valine participate in the energy regulation pathways of pantothenate and CoA biosynthesis. Pantothenic acid, a B5 vitamin, participates in many metabolic reactions by synthesizing coenzyme A (CoA) to provide energy for the body (de Villiers et al., 2013). In addition, studies have shown that the energy production of colonic cells in patients with UC is reduced and that the conversion of bound pantothenic acid to CoA in the diseased mucosa is blocked, resulting in increased pantothenic acid serum levels associated with UC. Furthermore, L-valine is an essential amino acid that cooperates with pantothenic acid to regulate the pantothenate and CoA biosynthesis pathways. Previous studies have confirmed that compared with healthy people, the level of L-valine, a serum metabolite, is decreased in UC patients, which may be related to the downregulation of tryptophan hydroxylase in the rectal tissue of UC patients, thus leading to intestinal function disorder (Schicho et al., 2012). However, after the TNBS-induced UC rats were treated with LZF, the levels of pantothenic acid and L-valine were restored to normal, indicating that LZF profoundly regulates the damaged energy metabolism pathway and provides an important guarantee for other metabolic activities.

Nicotinamide is the key precursor of nicotinamide adenine dinucleotide (NAD^+^). Nicotinamide is mainly absorbed through the gastrointestinal mucosa and plays an important role in cellular energy metabolism through the synthesis of NAD^+^. Recent studies have proved that nicotinamide also plays an important role in balancing intestinal dysfunction by regulating the inflammatory response, regulating the balance of the intestinal environment, and maintaining intestinal health (Shastri et al., 2020). Moreover, NAD^+^ generally regulates GPR109A and silent information regulator 2-related enzyme 1-mediated deacetylation to reduce the release of pro-inflammatory cytokines, including IL-1β, TNF-α, and NO (Chen et al., 2018). GPR109A and SIRT1 were widely expressed in colonic epithelial cells. In addition, SIRT1 is a positive regulator of NF-кB, which is considered to be an important transcriptional factor in the production of pro-inflammatory cytokines (Ma et al., 2016). In this study, the colonic epithelial mucosa of TNBS-induced UC rats was damaged, and nicotinamide was more likely to enter the circulatory system and continuously accumulate in the blood vessels. The metabolism of nicotinic acid and nicotinamide is supersaturated, resulting in the inactivation of two important enzymes, NAMPT and NMNAT. However, after treatment with LZF, the content of nicotinamide in serum decreased, indicating that LZF mediates the expression of GPR109A and SIRT1 in colonic epithelial cells by activating the enzymes NAMPT and NMNAT, thus playing a regulatory role in the interrupted metabolism of nicotinic acid and nicotinamide. This could provide sufficient energy for lipid and amino acid metabolism, especially it could repair the intestinal mucosal barrier function and improve the inflammatory response.

During treatment in the UC rat model, two metabolic pathways were closely related to the inflammatory response: primary bile acid biosynthesis and glycerophospholipid metabolism. Cholic acid (CA) is a primary bile acid (BA) first catalyzed from cholesterol in liver, and it facilitates the digestion and absorption of dietary lipids and the fat-soluble vitamins A, D, E, and K into the intestine. Then the conjugation of CA to taurine by CoA synthetase forms taurocholic acid. Approximately 5% of the BAs then escape into the colon, where gut commensal bacteria convert them into various intestinal secondary bile acids that are pivotal hormones in regulating cholesterol metabolism, energy balance, intestinal motility, and bacterial growth, as well as inflammation *via* several nuclear receptors such as farnesoid X receptor, pregnane X receptor, vitamin D receptor, and 1 G protein-coupled receptor (Zhou et al., 2014; Song et al., 2020). In a previous study, primary bile acid biosynthesis was remarkably perturbed in some UC disorders, and treatment with oral administration of CA or CDCA improved lipid adsorption and inhibited the synthesis of toxic bile acid intermediates in the intestinal microenvironment (Heubi et al., 2018). In our study of UC disease, treatment with LZF solution led to upregulated levels of cholic acid and taurocholic acid compared to the control group. This finding clarifies that UC disease with gut mucosal barrier and inflammation dysfunction can cause abnormal primary bile acid metabolism, which leads to bile acid enterohepatic circulation being blocked in the colon; the amount of bile acid returning to the liver was reduced, and the level of bile acid in serum decreased. These results indicate that LZF solution regulates the disrupted primary bile acid biosynthesis and regulates the BA-induced TGR5 signaling pathway with intestinal macrophages.

Glycerophospholipid metabolism participates in the regulation of inflammatory response through the synthesis of its derivative lysosomes. Glycerophospholipids are the structural components of cell membranes and contain many derivatives of glycerophosphoric acid, including lysophosphatidylcholines (LysoPCs) and lysophosphatidylethanolamines (LysoPEs). In general, LysoPCs have strong surface activity which can rupture red blood cells, changing their permeability and causing hemolysis or cell necrosis, and release histamine, serotonin, and epinephrine, stimulating a series of complex pathological reactions at the intestinal barrier as well as pro-inflammatory effects. However, unsaturated LysoPCs, such as LysoPC (15:0) and LysoPC (16:1), are potentially protective factors in inflammation, while saturated LysoPCs, such as LysoPC (18:0) and LysoPC (18:1), have been shown to promote proinflammatory cytokine release and exacerbate the inflammatory process (Jia et al., 2018; Guan et al., 2020). Moreover, previous studies have confirmed the presence of disturbed glycerophospholipids in patients and animals with UC (Wang et al., 2016). For example, dysregulation of the intestinal flora in IBD-model animals may lead to downregulation of glycerophospholipid synthesis, and the number of LysoPCs and LysoPEs in their serum has also been found to be reduced (Lu et al., 2012). In our study, LysoPC (15:0), LysoPC (16:1), and LysoPE (18:0) were upregulated upon LZF treatment in TNBS-induced UC, compared to the model group. These results are consistent with the favorable effects of unsaturated LysoPCs on the intestinal barrier and anti-inflammatory properties and indicate that some LysoPCs play roles in preventing mucosal inflammation to inhibit pivotal pro-inflammatory factors including IL-8, NO, and TNF-α in UC disease.

A previous study has shown the intestinal epithelium to have increased hypoxic stress and acutely increased energy requirements in the inflammation response of UC rats after induction with DSS, a metabolic stressor of epithelial cells (Turer et al., 2017). Creatine, an amino acid, is synthesized from three amino acids (glycine, arginine, and methionine), then exported from the liver to blood vessels (Brosnan and Brosnan, 2016). Creatine is interconverted to phosphocreatine and ADP in tissue cells with a rapid, high demand for creatine kinase and ATP. Therefore, the requirement for rapid ATP replenishment for amino acid metabolism *in vivo* within the colonic mucosal barrier plays an essential role in regulating intestinal homeostasis and protecting against UC. In our study, the creatine level in the model group was lower than that in the control group due to insufficient ATP energy supply in TNBS-induced UC rats. We inferred that tissue uptake of creatine would be blocked, resulting in increased content and accumulation, and would further disrupt arginine and proline metabolism. With the treatment of LZF solution, which ameliorates altered metabolites and pathways to repair the colonic barrier function, creatine is downregulated by normal energy metabolism functions with high CoA levels and increased ATP synthesis in cells. These results indicate that amino acid metabolisms containing creatine play important biological roles in UC disease, directly or indirectly linking energy metabolism with intestinal homeostasis by inhibiting inflammation and reducing oxidative stress.

In summary, the experimental results indicate that LZF solution plays a crucial role in metabolites in the treatment of UC and the regulation of main pathways of energy metabolism, anti-inflammatory response metabolism, and amino acid metabolism. Although this study has successfully identified specific UC biomarkers using serum metabolomics combined with HPLC-QTOF-MS technology, it has certain limitations. First, all active differential metabolites using metabolomics combined with genomics and proteomics in serum, urine, feces, colon tissue, and liver tissue of rats should be screened and analyzed by GC-MS, NMR, or HPLC-QTOF-MS to further clarify the full biological effects and bioactive integrated mechanisms of LZF solution in the treatment of UC. Second, Western blot and PCR studies should be used to elucidate essential protein pathways and related factors. Third, we should demonstrate the interplay of the gut microbiota and colon mucosa in anti-inflammatory processes. Such in-depth studies will better provide approaches to understanding the key metabolites and comprehensive metabolic pathways of the mechanism of treatment of UC.

## 5 Conclusion

Based on LZF’s anti-UC effect , this study used metabolomics technology to study its possible metabolic pathway, which provided a basis for revealing the mechanism of LZF in the treatment of UC. LZF showed excellent anti-UC effects and significantly inhibited the disease characteristics of weight loss, increased DAI, colon shortening, and inflammatory infiltration of intestinal tissue in rats by TNBS, and the effect was dose-dependent. Metabolomics study found 14 potential biomarkers, involving 12 metabolic pathways, among which the most significant ones included nicotate and nicotinamide metabolism, glycerophospholipid metabolism, arginine and proline metabolism, primary bile acid biosynthesis, and pantothenate and CoA biosynthesis. Therefore, LZF improves UC by regulating amino acid metabolism, fat metabolism, and energy production.

## Data Availability

The original contributions presented in the study are included in the article/Supplementary Material;, further inquiries can be directed to the corresponding authors.
